# The Impact of Front-of-Package Label Design on Consumer Understanding of Nutrient Amounts

**DOI:** 10.3390/nu10111624

**Published:** 2018-11-02

**Authors:** Samantha Goodman, Lana Vanderlee, Rachel Acton, Syed Mahamad, David Hammond

**Affiliations:** School of Public Health & Health Systems, University of Waterloo, 200 University Ave. W., Waterloo, ON N2L 3G1, Canada; samantha.goodman@uwaterloo.ca (S.G.); lana.vanderlee@uwaterloo.ca (L.V.); rachel.acton@uwaterloo.ca (R.A.); smahamad@uwaterloo.ca (S.M.)

**Keywords:** diet, adult, front-of-package, nutrition label, experiment

## Abstract

A between-groups experiment examined the salience of front-of-package (FOP) symbols. Adults from Canada, the US, Australia, and the UK completed an online survey (*n* = 11,617). Respondents were randomized to view cereal boxes displaying one of 11 FOP label conditions for ‘high’ levels of sugar and saturated fat: control (no FOP symbol), red circle, red ‘stop sign’, magnifying glass, magnifying glass + exclamation mark, and ‘caution’ triangle + exclamation mark, plus each of these five conditions accompanied by a ‘high in’ text descriptor. Participants identified the amount of saturated fat and sugar in the product (‘low’/’moderate’/’high’). Participants were more likely to correctly identify the product as ‘high’ in saturated fat or sugar when shown the stop sign, triangle + exclamation mark, red circle, or magnifying glass + exclamation mark symbols incorporating ‘high in’ text (*p* < 0.01). The magnifying glass was the least effective symbol. The stop sign (37.7%) and triangle + exclamation mark (22.0%) were most frequently selected as the best symbol for indicating high nutrient amounts. Overall, FOP labels with ‘high in’ descriptions, red color and intuitive ‘warning’ symbols (e.g., stop signs, exclamation marks, ‘caution’ triangles) were more effective at communicating high levels of nutrients of public health concern in a time-limited environment.

## 1. Introduction

The global health burden from poor diets is increasing [1]. Frequent consumption of foods high in saturated fat, sugars, or sodium can lead to overweight or obesity, hypertension, and cardiovascular disease [2]. This is a concern in many countries, including in Canada, where approximately two thirds of the adult population have overweight or obesity [3,4].

Nutrition labels are a prominent population-level intervention for communicating nutrition information. Food labels are notable for their reach—more Canadians report using nutrition information from food labels on prepackaged foods than from any other source—as well as their timing of exposure at the point-of-sale and consumption [5,6,7,8]. To date, prepackaged foods in most countries display ingredient lists and quantitative information on nutrient amounts. In Canada, the Nutrition Facts table (NFt) on prepackaged foods represents a prominent, credible source of nutrition information [9,10,11,12,13,14,15,16,17]. Nevertheless, research demonstrates that consumers have difficulty understanding and applying the information provided in the NFt [18,19,20], including identifying whether nutrient amounts are ‘high’ or ‘low’ compared to daily guidelines [21,22].

Front-of-package (FOP) nutrition labels seek to provide simple nutrition information in a more accessible location than NFts, which are typically displayed on the back or side of packages. A wide variety of FOP systems have been developed, including ‘high in’ labels, which seek to identify foods high in nutrients of public health concern [23,24]. Chile was the first country to implement mandatory FOP labels that signaled ‘high’ levels of calories, sugar, saturated fat, and sodium. The Government of Canada has committed to implementing a similar ‘high in’ FOP system [25,26] to inform consumers and encourage the reformulation of prepackaged foods [25]. In their consultation to identify an appropriate FOP label design, Health Canada included four alternative approaches using different symbols to highlight ‘high’ levels of saturated fat, sodium, and sugar [27], including the use of triangles, circles, and a magnifying glass.

Research has identified several characteristics that increase the effectiveness of FOP systems, including the use of recognizable symbols that are easy to understand [28]. The use of color has also been shown to increase attention to FOP labels [29,30] and may help consumers to make healthier choices [31,32,33,34]. Simple directional text, such as ‘high in (nutrient)’ is supported by consumers and has been shown to increase understanding of the healthfulness of products [35,36]. FOP labels that combine color with simple text descriptors may also enhance understanding of nutrient amounts [37]. Collectively, these design features influence the salience of FOP labels—the extent to which they are noticed—which is an important component of effective product labels [38,39]. Several methods have been used to test the salience of FOP designs, including eye-tracking studies that examine how long consumers spend looking at various types of labels [40,41,42,43]. Other studies have used quantitative or qualitative survey approaches to explore consumers’ perceptions of the ‘noticeability’ of different FOP labels [27,44]. Experimental tasks that involve time constraints are a particularly effective test of FOP salience, given that the average consumer typically spends fewer than 10 s viewing and assessing a food product label on any given shopping trip [6,45]. The current study employed a between-groups experimental task to test the salience of different FOP labeling designs, including different symbols, colors, and text descriptors, and whether this translates to immediate understanding of nutrient levels. It was hypothesized that more salient (i.e., noticeable) labels would be associated with a greater understanding of nutrient levels.

## 2. Materials and Methods

The current study was conducted as part of the International Food Policy Study in Australia, Canada, the UK, and the USA. Data were collected via self-completed web-based surveys conducted in December 2017 with adults aged 18–64 years. Respondents were recruited through the Nielsen Consumer Insights Global Panel [46] and partner panels. Email invitations with unique survey access links were sent to a random sample of panelists within the specified age and country criteria; panelists known to be ineligible were not invited. Surveys were conducted in English, French, or Spanish (based on the panelist’s known language preference); the experimental task described below was administered in English surveys only. Mean survey time across countries was 33 min.

Respondents provided consent prior to completing the survey, and received remuneration in accordance with their panel’s usual incentive structure (e.g., points-based or monetary rewards, chances to win prizes). The study was reviewed by and received ethics clearance through a University of Waterloo Research Ethics Committee (ORE# 21460). A full description of the study methods and measures can be found in the study’s technical report [47].

**Figure 1:**
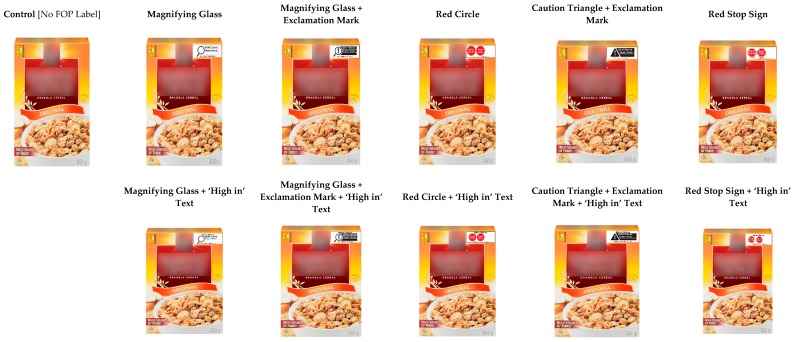
Images with front-of-package (FOP) labels displayed on screen for 4 s during experimental task. Note that brand names and logos have been blinded for publication but were shown to participants.

The current study presents findings from a between-groups experimental task that tested functional understanding of various FOP labels. Participants viewed an image of a cereal box displayed on the screen for 4 s. Cereal boxes displayed FOP labels for sugar and saturated fat according to the experimental condition to which the participant was randomized: (0) control (no FOP label); (1) red circle; (2) red stop sign; (3) magnifying glass; (4) magnifying glass + exclamation mark; and (5) ‘caution’ triangle + exclamation mark. Each of the five FOP symbols was also displayed with added ‘high in’ text, for a total of 11 experimental conditions (Figure 1). All label designs were modelled after early iterations of FOP symbols proposed by Health Canada [25,48,49].

After the image disappeared from the screen, participants were asked the following question for questions on saturated fat and sugar: “Is this amount of (saturated fat/sugar) in the product…? (‘Low’, ‘Moderate’, ‘High’, ‘Don’t know’, ‘Refuse’)”, with the correct response being ‘High’. For the purpose of this study, the product was considered to be ‘high’ in saturated fat and sugar.

**Figure 2:**
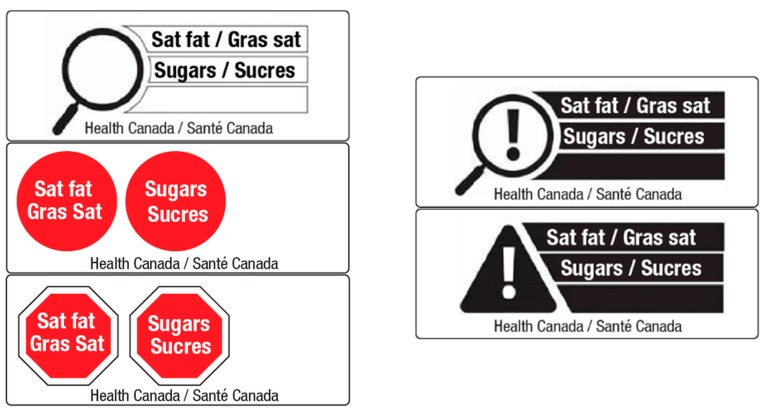
Front-of-package symbols * displayed on screen for the question, “Which is the best symbol for informing consumers that a product is ‘high in’ saturated fat and sugar?”. * Symbols presented in this question are identical to the five FOP symbol designs (i.e., without ‘high in’ text) tested in the experimental task (see Figure 1). Note that the government attribution (‘Health Canada’) was present for all participants, regardless of country of origin.

Following the experimental task, respondents viewed all five FOP designs and were asked, “Which is the best symbol for informing consumers that a product is ‘high in’ saturated fat and sugar?” Participants selected one of the five symbols, displayed in a random position on the screen (Figure 2).

## 3. Data Analysis

Analysis was conducted on a sample of 11,317 respondents who provided complete data on all measures. Chi-square tests were conducted to test for differences in sociodemographic factors (age, sex, education, and country) between experimental conditions. Separate logistic regression models were used to test for differences between conditions in the odds of responding correctly to the experimental questions on saturated fat and sugar; those who refused to answer were excluded. Models were adjusted for age group, sex, country, and highest education level (recoded to ‘Low’, ‘Middle’, or ‘High’ due to cross-country differences). Analyses were conducted using SPSS for Windows version 25.0 (IBM Corp., Armonk, NY, USA); *p* < 0.05 was considered significant.

## 4. Results

Sample characteristics are reported in Table 1. Chi-square tests revealed no significant differences in sociodemographic covariates between experimental conditions. For demographic data by country, see Table S1 (Supplementary Material).

### 4.1. Functional Understanding of FOP Labels (Experimental Task)

Table 2 shows the percentage of correct responses to the ‘high in’ saturated fat and sugar questions by experimental condition. In all cases, respondents shown the no-FOP control condition performed more poorly than those shown FOP symbols. Across conditions, responses to the question were as follow: Saturated fat: ‘Low’ (24.5%), ‘Moderate’ (26.1%), ‘High’ (7.9%), Don’t know (40.9%), Refuse (0.5%); sugar: ‘Low’ (15.7%), ‘Moderate’ (29.5%), ‘High’ (15.1%), Don’t know (39.2%), Refuse (0.5%).

Table 3 displays the results of the logistic regression (main effects model) which examined the effect of the various FOP label designs compared to the no-FOP control. For additional pairwise contrasts between FOP labels, see Table S2 (Supplementary Material). Participants who viewed the red stop sign, caution triangle + exclamation mark, red circle, or magnifying glass + exclamation mark FOP symbols incorporating ‘high in’ text were more likely to correctly identify the cereal as high in saturated fat and sugar compared to those who saw the no-FOP control. Notably, the red stop sign was effective even without ‘high in’ text. In contrast, those who saw the magnifying glass FOP labels had the lowest odds of responding correctly. Across all FOP designs, respondents who viewed labels with ‘high in’ text had greater odds of responding correctly: when ‘high in’ text was absent, the red circle and magnifying glass + exclamation mark symbols no longer significantly outperformed the control.

Younger (18–24 year-old) respondents had a higher likelihood of responding correctly compared to the oldest age groups. Those with a high level of education were significantly more likely to respond correctly compared to those with a low level. Finally, compared to those from the USA, respondents from Canada and the UK had a higher likelihood of responding correctly.

### 4.2. Perceived Effectiveness of FOP Designs

**Figure 3:**
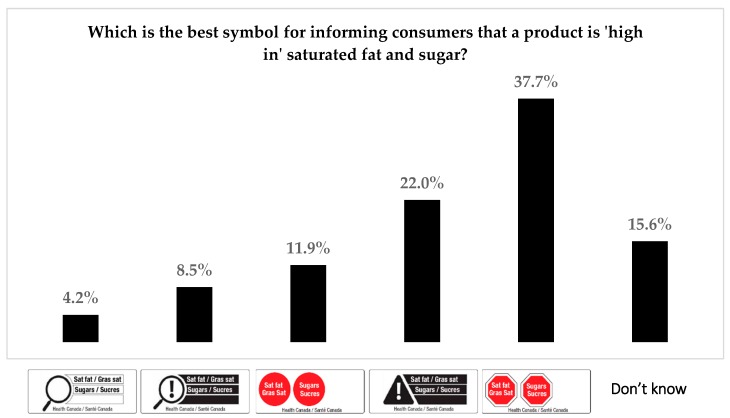
Responses to perceptions task: most informative front-of-package symbol (*n* = 11,617).

Figure 3 shows the percentage of respondents who selected each FOP symbol as being the best for informing consumers that a product is ‘high in’ saturated fat or sugar. The red stop sign (37.7%) and the triangle + exclamation mark (22.0%) were the most popular symbols; the magnifying glass (4.2%) was the least frequently selected.

## 5. Discussion

The design of FOP labels can have a measurable impact on consumer understanding even under highly restricted time limits. For all symbols tested except the magnifying glass, the presence of ‘high in’ text was associated with an increased likelihood of correctly identifying the cereal as being high in saturated fat and sugar. The inclusion of these descriptors may have been particularly important, given that all of the FOP symbols tested in the study would have been novel to participants.

The specific type of FOP symbol also influenced the likelihood that participants could identify a food as high in saturated fat or sugar. Compared to the control condition with no FOP label, participants who viewed the red stop sign with the ‘high in’ descriptor were almost three times as likely to report that the cereal was high in saturated fat and 1.6 times more likely to report that it was high in sugar. The red circle symbol and an exclamation mark placed within either a ‘caution’ triangle or a magnifying glass were also associated with more correct responses. Stop signs, ‘caution’ triangles, and exclamation marks are all familiar symbols that communicate warning. The intuitive meaning of these symbols is illustrated by the fact that for the question on saturated fat, the stop sign and caution triangle + exclamation mark outperformed the no-FOP control even in the absence of ‘high in’ text. In other words, the symbols themselves communicated the fundamental concept of warning. In contrast, the simple magnifying glass—which was recommended to Health Canada by the food industry [48]—was the least effective of all FOP label designs, likely because magnifying glasses have no intuitive association with high levels or the concept of ‘warning’. These findings are generally consistent with previous findings using behavioral purchase tasks, in which consumers who saw an exclamation mark FOP symbol made healthier purchases than those who saw other FOP labeling designs [50], as well as research indicating that Canadians prefer ‘high in’ FOP symbols featuring exclamation mark or triangle symbols [27]. In addition, the FOP symbols that performed best in the experimental task (red stop sign and triangle + exclamation mark) were also the most popular in the perceptions task. Although the effect of color was not systematically varied in the current study, the use of red color in the circle symbol may account for its superiority relative to the control condition, despite the fact that it did not incorporate a warning symbol [29,30].

Overall, the proportion of correct responses was fairly low (<20%); however, in this study design, the FOP labels were tested in a time-constrained setting to examine differences in the salience of warnings. Indeed, an important component of FOP systems is their use of symbols or images to engage consumers and communicate nutrient information ‘at a glance’ [28]. Therefore, the absolute level of correct responses is of secondary importance to the differences observed between conditions. If participants were provided additional time to view the warnings, the proportion of correct responses would likely have increased across the entire sample.

In examining the effects of sociodemographic covariates, we found that younger people were generally more likely to correctly understand FOP labels compared to their older counterparts, consistent with previous studies [51,52,53]. Overall, higher levels of education were also associated with a higher likelihood of responding correctly, consistent with previous research suggesting greater understanding of nutrition labels among more educated individuals [51,52,53]. Finally, the reason for the higher likelihood of responding correctly among respondents from Canada and the UK compared to the US is unknown, but may be a result of greater familiarity with FOP labeling systems and/or proposals in these countries. Given that the experiment tested FOP design elements being considered by Health Canada at the time, this finding also provides some reassurance of the utility of these labels in the Canadian market.

## 6. Strengths and Limitations

This study is subject to several limitations. First, the experiment was conducted online, rather than using actual products in a retail setting. However, the images used in the online survey were based on an actual product package and provided a reliable method for controlling viewing time. The length of viewing time was brief (4 s), which may have resulted in lower proportions of ‘correct’ responses than would have been the case if the image had remained on the screen for longer and/or while participants responded to questions. In addition to reflecting understanding, response rates may have therefore reflected participants’ recall ability; however, the randomized design would account for any such differences across experimental conditions. Within the experimental context, the absolute values of ‘correct’ responses are less meaningful than the relative differences between conditions; indeed, even within this highly constrained viewing time, the FOP designs led to substantial increases in correctly identifying foods high in nutrients of public health concern. The manipulation of FOP designs is also a potential limitation. The FOP designs were not systematically manipulated to examine the individual effect of color and symbol, or other differences between FOP labels, such as whether separate graphic symbols were used for each nutrient of concern (as in the red circle and stop sign symbols) or represented as a single symbol (as in the magnifying glass and triangle symbols). Future studies could test a full factorial design to isolate the effects of each design feature; however, the focus of the current study was to test specific proposals from Health Canada to inform a particular regulatory decision. As noted above, all the FOP symbols tested in the study were novel to participants; familiarity and understanding of FOP systems would likely be substantially greater if these systems were widely implemented on food products. This study only tested one product (cereal); although nutrient amounts do differ across products, we do not expect that the observed impact of specific label design elements would depend on product type. Finally, the use of a between-groups experimental design, diverse sample across four countries, and ‘objective’ measures of comprehension are all strengths of the current study.

## 7. Conclusions

Recognizable symbols (e.g., stop signs, ‘caution’ symbols, exclamation marks), (red) color, and simple text-based messages (e.g., ‘High in saturated fat’) are salient cues that help consumers quickly recognize high levels of nutrients of concern. This is important in the context of real shopping experiences, where purchasing decisions are made quickly.

## Figures and Tables

**Table 1:** Sample characteristics (*n* = 11,617).

Variable	% (*n*)
**Sex**	
Male	47.1% (5470)
Female	52.9% (6147)
**Age (years)**	
18–24	11.3% (1314)
25–30	33.1% (3849)
31–39	11.8% (1365)
40–49	12.5% (1455)
50–59	17.9% (2083)
60–64	13.4% (1551)
**Education level**	
Low	22.8% (2634)
Middle	27.7% (3202)
High	49.5% (5712)
**Country**	
USA	33.2% (3855)
Canada	7.0% (815)
Australia	28.4% (3302)
UK	31.4% (3645)

**Table 2:** Percentage of correct responses for each nutrient of concern by experimental condition * (*n* = 11,617).

FOP Experimental Condition	Saturated Fat	Sugar
**Control (no FOP Label)**	4.8%	12.3%
**Magnifying glass**		
No ‘high in’ text	5.3%	13.5%
‘High in’ text	6.5%	14.5%
**Magnifying glass + exclamation mark**		
No ‘high in’ text	5.6%	12.4%
‘High In’ text	8.9%	17.0%
**Red circle**		
No ‘high in’ text	5.9%	12.8%
‘High in’ text	10.3%	17.4%
**Triangle + exclamation mark**		
No ‘high in’ text	7.8%	15.0%
‘High in’ text	11.1%	18.5%
**Red stop sign**		
No ‘high in’ text	8.8%	15.8%
‘High in’ text	12.7%	18.0%

FOP, front-of-package. * Responses to question, “Is this amount of (saturated fat/sugar) in the product…?” (Low, Moderate, High, Don’t know, Refuse to answer). Correct response: ‘High’; ‘Don’t know’ coded as incorrect; ‘Refuse to answer’ excluded from analyses.

**Table 3:** Odds (OR, 95%CI) of a correct response * for each nutrient of concern (*n* = 11,617) **.

Variable	Saturated Fat	Sugar
**FOP label design**	***Χ*^2^ (10) = 98.50 ^c^**	***Χ*^2^ (10) = 44.33 ^c^**
Control (ref)	-	-
Magnifying glass	1.05 (0.71, 1.57)	1.07 (0.82, 1.39)
Magnifying glass + ‘High in’	1.41 (0.97, 2.05)	1.21 (0.94, 1.56)
Magnifying glass + Exclamation mark	1.17 (0.79, 1.72)	0.99 (0.76, 1.28)
Magnifying glass + Exclamation mark + ‘High in’	**1.94 (1.36, 2.76) ^c^**	**1.43 (1.12, 1.82) ^b^**
Red circle	1.22 (0.83, 1.79)	1.03 (0.79, 1.33)
Red circle + ‘High in’	**2.29 (1.61, 3.23) ^c^**	**1.48 (1.16, 1.89) ^b^**
Caution triangle + Exclamation mark	**1.68 (1.17, 2.41) ^b^**	1.21 (0.94, 1.56)
Caution triangle + Exclamation mark + ‘High in’	**2.51 (1.77, 3.55) ^c^**	**1.60 (1.25, 2.05) ^c^**
Red stop sign	**1.92 (1.34, 2.75) ^c^**	**1.34 (1.04, 1.72) ^a^**
Red stop sign + ‘High in’	**2.95 (2.10, 4.15) ^c^**	**1.59 (1.25, 2.03) ^c^**
**Sex**	***Χ*^2^ (1) = 0.23**	***Χ*^2^ (1) = 1.29**
Male (ref)	-	-
Female	1.04 (0.90, 1.19)	1.06 (0.96, 1.18)
**Age (years)**	***Χ*^2^ (5) = 41.62 ^c^**	***Χ*^2^ (5) = 18.10 ^b^**
18–24 (ref)	-	-
25–30	1.07 (0.85, 1.35)	**0.79 (0.67, 0.95) ^a^**
31–39	0.99 (0.75, 1.30)	0.87 (0.71, 1.07)
40–49	0.78 (0.59, 1.03)	**0.79 (0.64, 0.97) ^a^**
50–59	**0.63 (0.48, 0.83) ^b^**	**0.69 (0.57, 0.84) ^c^**
60–64	**0.56 (0.41, 0.76) ^c^**	**0.69 (0.56, 0.86) ^b^**
**Education**	***Χ*^2^ (2) = 6.33 ^a^**	***Χ*^2^ (2) = 22.43 ^c^**
Low (ref)	-	-
Middle	1.03 (0.85, 1.24)	1.00 (0.86, 1.16)
High	**1.26 (1.06, 1.49) ^a^**	**1.30 (1.13, 1.48) ^c^**
**Country**	***Χ*^2^ (3) = 64.98 ^c^**	***Χ*^2^ (3) = 190.20 ^c^**
USA (ref)	-	-
Canada	**2.74 (2.11, 3.55) ^c^**	**3.28 (2.70, 3.98) ^c^**
Australia	1.03 (0.85, 1.24)	0.99 (0.85, 1.14)
UK	**1.26 (1.06, 1.50) ^b^**	**1.55 (1.36, 1.77) ^c^**

95% CI, 95% confidence intervals; *Χ*^2^, chi-square; FOP, front-of-package; OR, odds ratio; ref, reference group. * Responses to question, “Is this amount of (saturated fat/sugar) in the product…? (Low, Moderate, High, Don’t know, Refuse to answer)”. Correct response: ‘High’; ‘Don’t know’ coded as incorrect; ‘Refuse to answer’ excluded from analyses ** *Χ*^2^ (*df*) reported for main effects; OR (95% CI) reported for pairwise contrasts. Significant effects are indicated in bold; reference categories are denoted with “-“; superscript letters ^a^, ^b^, and ^c^ indicate significance at *p* < 0.05, *p* < 0.01, and *p* < 0.001, respectively.

## Supplementary Materials

The following are available online at http://www.mdpi.com/2072-6643/10/11/1624/s1. Table S1: Socio-demographic characteristics (%, *n*) by country (*n* = 11,617), Table S2.: Odds (OR, 95%CI) of a correct response: Additional contrasts between FOP symbol designs (*n* = 11,617).
