# Supplementary material for: The Impact of Front-of-Package Label Design on Consumer Understanding of Nutrient Amounts
Source: Nutrients. 2018 Nov 2;10(11):1624. doi: 10.3390/nu10111624 (PMC6266389; doi:10.3390/nu10111624)
Supplement: Supplementary file 1 [file nutrients-10-01624-s001.pdf]

Table S1. Socio-demographic characteristics (%, n) by country (n=11,617).

Variable	USA (*n* = 3855)	Canada (*n* = 815) *	Australia (*n* = 3302)	UK (*n* = 3645)
**Sex**				
Male	48.7% (1879)	52.0% (424)	41.3% (1364)	49.5% (1803)
Female	51.3% (1976)	48.0% (391)	58.7% (1938)	50.5% (1842)
**Age (years)**				
18–24	7.4% (286)	0% (0)	15.6% (514)	14.1% (514)
25–30	42.9% (1652)	0% (0)	26.7% (882)	36.1% (1315)
31–39	9.2% (353)	18.0% (147)	11.7% (386)	13.1% (479)
40–49	9.9% (380)	22.2% (181)	12.8% (424)	12.9% (470)
50–59	16.0% (617)	33.6% (274)	19.3% (636)	15.3% (556)
60–64	14.7% (567)	26.1% (213)	13.9% (460)	8.5% (311)
**Education**				
Low	18.0% (690)	18.7% (152)	27.1% (890)	24.9% (902)
Middle	18.0% (690)	39.7% (323)	35.2% (1153)	28.7% (1036)
High	64.1% (2459)	41.6% (339)	37.7% (1236)	46.4% (1678)

*Participants aged 18–32 years from Canada were recruited from a separate study and did not complete the experimental question examined herein. For further details, please refer to: http://foodpolicystudy.com/methods/.

Table S2. Odds (OR, 95%CI) of a correct response*: additional contrasts between FOP symbol designs (*n* = 11,617).

FOP symbol design	Saturated Fat	Sugar
Red circle (ref)	---	---
Red circle + High in	**1.87 (1.35, 2.59)** [c]	**1.44 (1.13, 1.84)** [b]
Red stop sign	**1.58 (1.13, 2.20)** [b]	**1.30 (1.02, 1.67)** [a]
Red stop sign + High in	**2.42 (1.76, 3.32)** [c]	**1.55 (1.22, 1.97)** [c]
Magnifying glass + Exclamation mark	0.96 (0.66, 1.38)	0.96 (0.74, 1.24)
Magnifying glass + Exclamation mark + High in	**1.59 (1.14, 2.22)** [b]	**1.39 (1.09, 1.77)** [b]
Magnifying glass	0.86 (0.59, 1.26)	1.04 (0.80, 1.34)
Magnifying glass + High in	1.16 (0.81, 1.65)	1.18 (0.92, 1.51)
Caution triangle + Exclamation mark	1.38 (0.98, 1.94)	1.18 (0.92, 1.51)
Caution triangle + Exclamation mark + High in	**2.06 (1.49, 2.84)** [c]	**1.56 (1.22, 1.99)** [c]
Red circle + High in (ref)	---	---
Red circle	**0.54 (0.39, 0.74)** [c]	**0.69 (0.54, 0.89)** [b]
Red stop sign	0.84 (0.63, 1.13)	0.91 (0.72, 1.14)
Red stop sign + High in	1.30, (0.99, 1.70)	1.08 (0.86, 1.35)
Magnifying glass + Exclamation mark	**0.51 (0.37, 0.71)** [c]	**0.67 (0.52, 0.85)** [b]
Magnifying glass + Exclamation mark + High in	0.85 (0.64, 1.14)	0.96 (0.77, 1.21)
Magnifying glass	**0.46 (0.33, 0.65)** [c]	**0.72 (0.57, 0.92)** [b]
Magnifying glass + High in	**0.62 (0.45, 0.85)** [b]	0.82 (0.65, 1.04)
Caution triangle + Exclamation mark	**0.74 (0.54, 0.99)** [a]	0.82 (0.65, 1.04)
Caution triangle + Exclamation mark + High in	1.10 (0.83, 1.46)	1.08 (0.86, 1.36)
Red stop sign (ref)	**---**	---
Red circle	**0.64 (0.45, 0.89)** [b]	**0.77 (0.60, 0.98)** [a]
Red circle + High in	1.19 (0.88, 1.60)	1.11 (0.87, 1.40)

Red stop sign + High in	**1.54 (1.16, 2.04)** [b]	1.19 (0.94, 1.50)
Magnifying glass + Exclamation mark	**0.61 (0.43, 0.86)** [b]	**0.73 (0.57, 0.94)** [a]
Magnifying glass + Exclamation mark + High in	1.01 (0.75, 1.36)	1.06 (0.84, 1.34)
Magnifying glass	**0.55 (0.38, 0.78)** [b]	0.80 (0.62, 1.02)
Magnifying glass + High in	0.73 (0.53, 1.02)	0.90 (0.71, 1.15)
Caution triangle + Exclamation mark	0.87 (0.64, 1.19)	0.91 (0.71, 1.15)
Caution triangle + Exclamation mark + High in	1.31 (0.97, 1.75)	1.20 (0.95, 1.51)
Red stop sign + High in (ref)	---	---
Red circle	**0.41 (0.30, 0.57)** [c]	**0.65 (0.51, 0.82)** [c]
Red circle + High in	0.77 (0.59, 1.02)	0.93 (0.74, 1.17)
Red stop sign	**0.65 (0.49, 0.86)** [b]	0.84 (0.67, 1.06)
Magnifying glass + Exclamation mark	**0.40 (0.29, 0.55)** [c]	**0.62 (0.48, 0.79)** [c]
Magnifying glass + Exclamation mark + High in	**0.66 (0.50, 0.87)** [b]	0.90 (0.71, 1.13)
Magnifying glass	**0.36 (0.26, 0.50)** [c]	**0.67 (0.53, 0.86)** [b]
Magnifying glass + High in	**0.48 (0.35, 0.65)** [c]	**0.76 (0.60, 0.96)** [a]
Caution triangle + Exclamation mark	**0.57 (0.43, 0.76)** [c]	**0.76 (0.60, 0.96)** [a]
Caution triangle + Exclamation mark + High in	0.85 (0.65, 1.11)	1.01 (0.80, 1.26)
*Magnifying glass + Exclamation ma*rk *(ref)*	---	---
Red circle	1.05 (0.72, 0.51)	1.04 (0.81, 1.35)
Red circle + High in	**1.96 (1.40, 2.73)** [c]	**1.50 (1.18, 1.93)** [b]
Red stop sign	**1.65 (1.17, 2.32)** [b]	**1.36 (1.06, 1.75)** [a]
Red stop sign + High in	**2.53 (1.83, 3.50)** [c]	**1.62 (1.26, 2.07)** [c]
Magnifying glass + Exclamation mark + High in	**1.66 (1.18, 2.33)** [b]	**1.45 (1.13, 1.85)** [b]
Magnifying glass	0.90 (0.61, 1.33)	1.09 (0.84, 1.41)
Magnifying glass + High in	1.21 (0.84, 1.73)	1.23 (0.96, 1.59)
Caution triangle + Exclamation mark	**1.44 (1.02, 2.04)** [a]	1.23 (0.96, 1.59)
Caution triangle + Exclamation mark + High in	**2.15 (1.54, 2.99)** [c]	**1.63 (1.27, 2.08)** [c]
Magnifying glass + Exclamation mark + High in (ref)	---	---
Red circle	**0.63 (0.45, 0.88)** [b]	**0.72 (0.57, 0.92)** [b]
Red circle + High in	1.18 (0.88, 1.58)	1.04 (0.83, 1.31)
Red stop sign	0.99 (0.73, 1.34)	0.94 (0.74, 1.19)
Red stop sign + High in	**1.52 (1.15, 2.02)** [b]	1.12 (0.89, 1.40)
Magnifying glass + Exclamation mark	**0.60 (0.43, 0.85)** [b]	**0.69 (0.54, 0.88)** [b]
Magnifying glass	**0.54 (0.38, 0.77)** [b]	**0.75 (0.59, 0.96)** [a]
Magnifying glass + High in	0.73 (0.53, 1.01)	0.85 (0.67, 1.08)
Caution triangle + Exclamation mark	0.87 (0.64, 1.18)	0.85 (0.67, 1.08)
Caution triangle + Exclamation mark + High in	1.29 (0.97, 1.73)	1.12 (0.90, 1.41)
Magnifying glass (ref)	---	---
Red circle	1.16 (0.79, 1.70)	0.96 (0.74, 1.24)
Red circle + High in	**2.17 (1.54, 3.06)** [c]	**1.39 (1.09, 1.77)** [b]
Red stop sign	**1.83 (1.28, 2.60)** [b]	1.26 (0.98, 1.61)
Red stop sign + High in	**2.81 (2.01, 3.93)** [c]	**1.49 (1.17, 1.90)** [b]
Magnifying glass + Exclamation mark	1.11 (0.75, 1.63)	0.92 (0.71, 1.20)
Magnifying glass + Exclamation mark + High in	**1.84 (1.30, 2.62)** [b]	**1.34 (1.05, 1.70)** [a]
Magnifying glass + High in	1.34 (0.93, 1.94)	1.13 (0.88, 1.46)
Caution triangle + Exclamation mark	**1.60 (1.12, 2.28)** [a]	1.14 (0.89, 1.46)
Caution triangle + Exclamation mark + High in	**2.38 (1.69, 3.36)** [c]	**1.50 (1.18, 1.91)** [b]
Magnifying glass + High in (ref)	---	---

Red circle	0.87 (0.61, 1.23)	0.85 (0.66, 1.09)
Red circle + High in	**1.62 (1.18, 2.22)** [b]	1.22 (0.97, 1.55)
Red stop sign	1.36 (0.98, 1.89)	1.11 (0.87, 1.41)
Red stop sign + High in	**2.09 (1.54, 2.84)** [c]	**1.31 (1.04, 1.66)** [a]
Magnifying glass + Exclamation mark	0.83 (0.58, 1.19)	0.81 (0.63, 1.05)
Magnifying glass + Exclamation mark + High in	1.37 (0.99, 1.90)	1.18 (0.93, 1.49)
Magnifying glass	0.75 (0.52, 1.08)	0.88 (0.69, 1.13)
Caution triangle + Exclamation mark	1.19 (0.86, 1.66)	1.00 (0.79, 1.28)
Caution triangle + Exclamation mark + High in	**1.78 (1.30, 2.43)** [c]	**1.32 (1.05, 1.67)** [a]
Caution triangle + Exclamation mark (ref)	---	---
Red circle	0.73 (0.52, 1.02)	0.85 (0.66, 1.09)
Red circle + High in	**1.36 (1.01, 1.84)** [a]	1.22 (0.97, 1.54)
Red stop sign	1.15 (0.84, 1.56)	1.10 (0.87, 1.40)
Red stop sign + High in	**1.76 (1.32, 2.35)** [c]	**1.31 (1.04, 1.66)** [a]
Magnifying glass + Exclamation mark	**0.70 (0.49, 0.98)** [a]	0.81 (0.63, 1.04)
Magnifying glass + Exclamation mark + High in	1.16 (0.85, 1.57)	1.18 (0.93, 1.49)
Magnifying glass	**0.63 (0.44, 0.90)** [b]	0.88 (0.69, 1.13)
Magnifying glass + High in	0.84 (0.60, 1.17)	1.00 (0.78, 1.27)
Caution triangle + Exclamation mark + High in	**1.49 (1.11, 2.01)** [b]	**1.32 (1.05, 1.67)** [a]
Caution triangle + Exclamation mark + High in (ref)	---	---
Red circle	1.16 (0.79, 1.70)	0.96 (0.74, 1.24)
Red circle + High in	**2.17 (1.54, 3.06)** [c]	**1.39 (1.09, 1.77)** [b]
Red stop sign	**1.83 (1.28, 2.60)**	1.26 (0.98, 1.61)
Red stop sign + High in	**2.81 (2.01, 3.93)** [c]	**1.49 (1.17, 1.90)** [b]
Magnifying glass + Exclamation mark	1.11 (0.75, 1.63)	0.92 (0.71, 1.20)
Magnifying glass + Exclamation mark + High in	**1.84 (1.30, 2.62)** [b]	**1.34 (1.05, 1.70)** [a]
Magnifying glass	1.34 (0.93, 1.94)	1.13 (0.88, 1.46)
Magnifying glass + High in	**1.60 (1.12, 2.28)** [a]	1.14 (0.89, 1.46)
Caution triangle + Exclamation mark	**2.38 (1.69, 3.36)** [c]	**1.50 (1.18, 1.91)** [b]

95%CI, 95% confidence intervals; FOP, front-of-package; OR, odds ratio; ref, reference group.

*Responses to question, "Is this amount of [saturated fat/sugar] in the product? (Low, Moderate, High, Don't know, Refuse to answer)". Correct responses: 'High'; 'Don't know' coded as incorrect; 'Refuse to answer' excluded from analyses. Significant effects indicated in bold; superscript letters a, b and c indicate significance at $p < 0.05$, $p \leq 0.01$ and $p \leq 0.001$, respectively. All models were adjusted for sex, age group, country and education level.
